# Estimation of Incidence of Typhoid and Paratyphoid Fever in Vientiane, Lao People’s Democratic Republic

**DOI:** 10.4269/ajtmh.19-0634

**Published:** 2020-03-02

**Authors:** Phetsavanh Chanthavilay, Mayfong Mayxay, Phouthapanya Xongmixay, Tamalee Roberts, Sayaphet Rattanavong, Manivanh Vongsouvath, Paul N. Newton, John A. Crump

**Affiliations:** 1 Institute of Research and Education Development, University of Health Sciences, Vientiane, Lao People’s Democratic Republic;; 2 Lao-Oxford-Mahosot Hospital-Wellcome Trust Research Unit (LOMWRU), Microbiology Laboratory, Vientiane, Lao People’s Democratic Republic;; 3 Nuffield Department of Medicine, Centre for Tropical Medicine and Global Health, Churchill Hospital, Oxford, United Kingdom;; 4 Centre for International Health, University of Otago, Dunedin, New Zealand

## Abstract

Typhoid conjugate vaccines represent a new tool for typhoid control. However, incidence data are needed to inform decisions about introduction. We sought to estimate typhoid and paratyphoid fever incidence in Vientiane, the capital and largest city of the Lao People’s Democratic Republic (Lao PDR). We did a representative cluster survey of health-seeking behavior for fever in Vientiane from January 15, 2019 through January 26, 2019. Multipliers derived from the survey were applied to data from *Salmonella* Typhi and *Salmonella* Paratyphi A bloodstream infection surveillance from Mahosot Hospital, Vientiane, for the period of January 1, 2015 through December 31, 2017, to estimate enteric fever incidence. A total of 336 households representing 1,740 persons were enrolled in the healthcare utilization survey, and multipliers were derived based on responses to questions about healthcare seeking in the event of febrile illness. Of 7,997 Vientiane residents receiving blood cultures over the 2-year surveillance period at Mahosot Hospital, we identified 16 (0.2%) with *Salmonella* Typhi and six (< 0.1%) with *Salmonella* Paratyphi A bloodstream infection. After applying multipliers, we estimated that the annual incidence of typhoid was 4.7 per 100,000 persons and paratyphoid was 0.5 per 100,000 persons. During the study period, the incidence of typhoid and paratyphoid fever was low in Vientiane. Ongoing surveillance is warranted to identify increases in future years. Similar studies elsewhere in the Lao PDR would be useful to understand the wider enteric fever situation in the country.

## INTRODUCTION

*Salmonella enterica* serovars Typhi and ‘most often’ Paratyphi A cause typhoid and paratyphoid fever, collectively known as enteric fever. Both pathogens are restricted to humans and are transmitted predominantly by water and food contaminated by human feces. In 2017, typhoid and paratyphoid fever were estimated to cause 10.9 and 3.4 million illnesses^1^ and 116,800 and 19,100 deaths, respectively.^2^ In Southeast Asia in 2017, enteric fever incidence was estimated to be 219.8 per 100,000 populations and associated with 877,200 disability-adjusted life-year lost.^3^ As with other countries in Southeast Asia,^4^ *Salmonella* Typhi has been identified as a leading cause of community-onset bloodstream infection in the Lao People’s Democratic Republic (Lao PDR), not only in the capital of Vientiane^5^ but also in Luang Namtha in the northwest and Salavan in the south.^6^ Furthermore, multiple-drug resistance has been identified among *Salmonella* Typhi isolates in Lao PDR.^6^

Although hospital-based studies of community-onset bloodstream infection are useful to estimate the prevalence of common causes of severe febrile illness and to guide empiric management, estimates of disease incidence are a critical building block of burden of disease estimates.^7^ However, measuring disease incidence is challenging. Estimates based on sentinel surveillance at one or more healthcare facilities alone are likely to underestimate cases, identifying only those who are able to access the facility for care. Population-based studies are resource intensive, ideally involving frequent visits to all households in a large cohort to identify persons with fever to either collect a blood culture at home or actively refering such persons to a center where blood culture is systematically provided.^8^ Over the past two decades, hybrid surveillance methods, also known as “multiplier studies,” combining sentinel surveillance with healthcare utilization surveys to adjust for under-ascertainment have been developed and applied to provide a practical means of estimating infectious disease incidence.^8,9^ In 2017, the WHO Strategic Advisory Group of Experts on immunization recommended a routine use of typhoid conjugate vaccine in infants and children aged > 6 months in typhoid-endemic countries.^10^ National decisions about typhoid conjugate vaccine introduction can be informed by robust typhoid incidence data. However, such data are unavailable for the Lao PDR. In a parallel study, we used the data from blood cultures received at Mahosot Hospital, Vientiane, from diverse parts of Laos, to provide a minimal estimate of inpatient culture-positive typhoid incidence, with the most recent annual incidence in 2018 of 0.59 cases per 100,000 persons in the community.^11^ However, because of limitations of access to Mahosot Hospital from remote areas and few blood culture services outside Vientiane, this estimate of annual incidence of hospitalized typhoid is likely to be a substantial underestimate of the true incidence of the disease in the community.

To develop an estimate of typhoid and paratyphoid fever incidence at the community level in the Lao PDR, we combined sentinel surveillance data from Mahosot Hospital with a healthcare utilization survey in the hospital’s main catchment area of Vientiane. The goal was to provide contemporary estimates of disease incidence to inform typhoid prevention and control strategies.

## MATERIALS AND METHODS

### Study location.

With a population of 820,940, Vientiane is both the capital and the most populous city in Lao PDR.^12^ Mahosot Hospital is a 450-bed public referral and teaching hospital providing outpatient and inpatient services to people of all ages living in Vientiane and beyond, including urban, semi-urban, and rural areas. A sister study based in Yangon, Myanmar, has been published elsewhere.^13^

### Healthcare utilization survey.

#### Sample size.

The sample size estimation for the healthcare utilization survey was based on the WHO vaccination coverage cluster survey method.^14^ Assumptions included a single stratum to estimate healthcare utilization for the catchment area of hospital, an effective sample size of 103, *m* = 5 respondents per cluster, and an intra-cluster correlation coefficient of 0.33 to yield a design effect of 2.32. We assumed that 100% of households had the potential to yield at least one participant of any age and that 20% of households would have no one at home after three visits or would refuse to participate, for an inflation factor of 1.25. These assumptions yielded a total target of 239 respondents, requiring visit to 299 households to obtain 239 completed questionnaires accounting for nonresponse. These households were sought in 48 clusters, with seven households to visit per cluster per site.

#### Household selection.

Household selection was carried out by two-stage cluster survey. In the first stage, 48 villages were selected proportional to the population size from 485 villages of Vientaine city based on the Lao PDR Population and Housing Census 2015.^12^ In the second stage, seven households were selected by simple random sampling from village household lists obtained from the village head.

#### Design and administration of the survey.

The healthcare utilization survey was carried out from January 15, 2019 through January 26, 2019. After obtaining written informed consent, members of the study team administered the questionnaire to the heads of seven households in each village. The questionnaire was based on those widely used in other surveys of healthcare utilization^13,15,16^ and included questions about demographics, socioeconomic status, and healthcare-seeking behavior. Healthcare-seeking questions asked separately about usual healthcare-seeking behavior in the event of fever for < 3 days and ≥ 3 days duration in age-groups < 5 years, 5–< 12 years, and ≥ 12 years, as well as actual healthcare-seeking behavior of any individual household members experiencing fever in the past 3 months. Choices included Mahosot Hospital, other public and private hospitals, and health centers in Vientiane as well as private clinics, grocers, mobile drug vendors, village health volunteers, traditional healers, self-treatment, and not seeking care.

### Surveillance for community-acquired bloodstream infections.

As a part of ongoing bloodstream infection surveillance, Mahosot Hospital provides blood culture free of charge to all patients presenting for admission with body temperature > 37.5°C or < 36.5°C, 24 hours per day and 7 days per week. As soon as possible after presentation, before the initiation of antimicrobial therapy, and following cleansing of the skin with povidone–iodine solution, venous blood was drawn for blood culture from each patient. The volume of blood inoculated into each bottle to achieve a blood-to-broth ratio of 1:10 was 1 mL from children aged < 1 year, 2 mL from children aged 1–15 years, and 5 mL from those aged > 15 years.

Blood was inoculated into a tryptic hydrolysate of casein and soy peptone broth blood culture bottle (Pharmaceutical Factory No. 2, Vientiane, Lao PDR). Inoculated bottles were sent to the microbiology laboratory, Mahosot Hospital, for incubation, isolation, identification, and antimicrobial susceptibility testing as previously described.^5^ For this analysis, we extracted data from an ongoing bloodstream infections surveillance database for the period January 1, 2015 through December 31, 2017 along with relevant demographic data, such as the participant’s village of residence. Isolation of the same organism from multiple blood cultures during the same admission was classified as a single episode of bacteremia.^17,18^

#### Laboratory methods.

Inoculated blood culture bottles were incubated at 35–37°C in air for 7 days and examined daily for signs of bacterial growth. Turbid bottles were subcultured onto goat blood and chocolate agar. ‘Blind’ subculture was performed on day 1 and 7 postinoculation. Bacteria were identified using standard microbiological techniques, API 20E (bioMerieux, Marcy L’Etoile, France) and were confirmed as *Salmonella* Typhi or *Salmonella* Paratyphi A by agglutination with poly-O, O2, Hd, and Vi antisera (Becton Dickinson Laboratories, Franklin Lakes, NJ) and group-specific antisera (A & A reagents, Bangkok, Thailand). Antimicrobial susceptibility testing was by disk diffusion on Mueller–Hinton agar (Oxoid, Basel, Switzerland) following U.S. Clinical and Laboratory Standards Institute guidelines,^19^ and minimum inhibitory concentrations were determined by using the Etest (AB Biodisk, Solna, Sweden).

### Incidence calculation.

**Figure 1.**
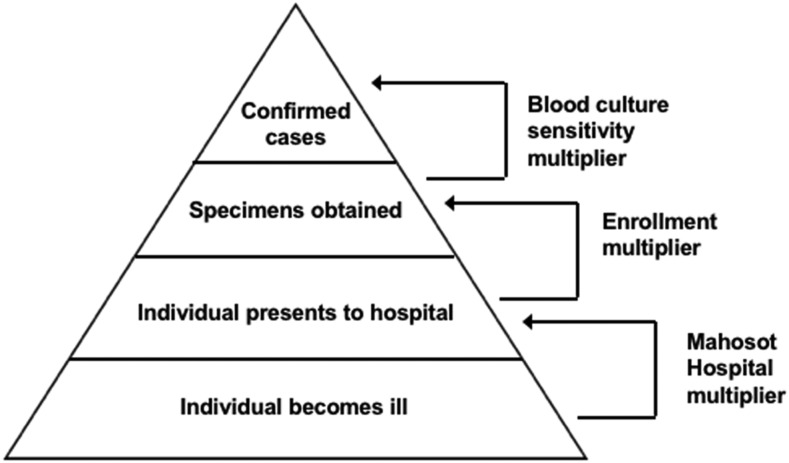
Surveillance pyramid showing multipliers used to account for incomplete case detection. Mofidied from Crump et al.^8^

We estimated incidence by age-group and overall using age-specific multipliers derived from the healthcare utilization survey and Mahosot Hospital bloodstream infection surveillance. *Salmonella* Typhi and *Salmonella* Paratyphi A bloodstream infections for the 3-year surveillance period were calculated as a mean annual number. Multipliers accounted for persons with typhoid and paratyphoid fever who would potentially be missed through stages of reporting, including healthcare facility choice for fever; proportion of febrile patients seen at Mahosot Hospital receiving blood culture; and blood culture sensitivity for the diagnosis of enteric fever (Figure 1). Multipliers were multiplicative, inverse to the relevant proportions. The ‘Mahosot Hospital multiplier’ accounted for patients with fever who would seek care at other healthcare facilities. The culture sensitivity multiplier was the same for all age-groups. The age-specific Mahosot multipliers included children aged 0–< 5 years, 5–< 12 years, ≥ 12 years, and any age. The Mahosot Hospital mulplier was derived from the following question: ‘Where would you usually seek health care if a household member had fever ≥ 3 days duration?’ We selected the first and second choice responses to ‘fever ≥ 3 days duration’ as most representative of where patients sufficiently ill requiring admission would seek care. We validated responses to questions about usual healthcare-seeking against actual healthcare seeking of household members who had fever ≥ 3 days in the past 3 months.

We calculated the ‘enrollment multiplier’ to account for patients eligible for blood culture, but not enrolled in surveillance for any reason. To estimate the enrollment multiplier, we reviewed febrile admissions to Mahosot Hospital for the period of January 1, 2019 through June 30, 2019, representing a dry and rainy season of Lao PDR. The enrollment multiplier was calculated as the inverse of the proportion of febrile patients receiving a blood culture. Because enrollment took place 24 hours per day, 7 days per week, there was no need to account for time periods when enrollment did not take place. We calculated a blood culture sensitivity multiplier reflecting the sensitivity of blood culture for the diagnosis of typhoid and paratyphoid fever compared with bone marrow aspirate culture.^20,21^

### Sensitivity analyses.

We conducted one-way sensitivity analyses to determine the degree of uncertainty of our incidence estimates using 95% CIs for the hospital multipliers. Upper and lower bound 95% CI was calculated using binomial exact distribution.

### Statistical analyses.

Health-seeking behavior survey data entry and incidence calculations were performed using Microsoft Excel 2010 (Microsoft Corporation, Redmond, WA) spreadsheets. Other analyses were performed using STATA, version 12 (STATA-Corp, College Station, TX). All *P* values are two-sided, and statistical significance was set at *P* < 0.05. Because of low numbers of typhoid and paratyphoid cases at Mahosot Hospital, 3-year cumulative numbers of confirmed cases were used to estimate the yearly incidence.

### Research ethics.

The study was approved by the Lao National Ethics Committee for Health Research (NIMR1HQ/R.8cNo1. 11/283) and the University of Otago Human Ethics Committee, New Zealand.

## RESULTS

### Healthcare utilization survey.

Among the 48 selected villages, the median (range) distance from the village midpoint to Mahosot Hospital was 12 (1, 59) km. We enrolled 336 households, including 1,740 household members. Among selected households, 18 (5.3%) were not available to participate after three return visits and were replaced by another randomly selected household. The median (range) age of household members was 20 (< 1, 109) years, and 923 (53.1%) were female. The median (range) age of household head respondents was 53 (30, 86) years and 41 (26.3%) years were female. Of households, 336 (100%) had at least one member aged ≥ 12 years, 117 (34.8%) households had at least one member aged from 5 to < 12 years, and 66 (19.6%) households had at least one member aged < 5 years.

### Multiplier derivation.

Of heads of household interviewed, 162 (48.2%) of 336 reported that any household member would use Mahosot Hospital in the event of fever ≥ 3 days duration: 16 (24.2%) of 66 for household members aged 0 to < 5 years; 36 (30.7%) of 117 for household members aged ≥ 5 to < 12 years; and 161 (47.9%) of 336 for household members aged ≥ 12 years. These proportions correspond to Mahosot Hospital multipliers of 1.6, 4.1, 3.2, and 2.1 for all ages, ages 0 through < 5 years, ≥ 5 to < 12 years, and ≥ 12 years, respectively (Table 1). Of 953 patients admitted to Mahosot Hospital with fever January 1, 2019 through June 30, 2019, in age-groups of all ages, 0 through < 5 years, ≥ 5 to < 12 years, and ≥ 12 years, 678 (71.1%), 127 (46.5%), 84 (67.2%), and 467 (84.1%) received a blood culture, respectively, yielding enrollment multipliers of 1.4, 2.1, 1.5, and 1.2, respectively. The proportion receiving a blood culture was 53.0% among those aged < 12 years and 84.1% among those aged ≥ 12 years (*P* < 0.001). We used a blood culture sensitivity multiplier of 2.0 to reflect the sensitivity of a single blood culture for the diagnosis of typhoid and paratyphoid fever compared with culture of aspirated bone marrow.^20,21^

**Table 1:** Enteric fever incidence estimates, Vientiane, Lao PDR, 2015–2017

	Age-group (years)	Mahosot Hospital–confirmed cases, 2015–17	Sensitivity multiplier	Mahosot Hospital multiplier	Enrollment multiplier	Cases, 2015–17	Annual cases	Population	Incidence per 100,000 per year
Typhoid fever	0 to < 5	0	2	4.1	2.1	–	–	65,904	0.0
	5 to < 12	2	2	3.3	1.5	19.5	6.5	87,749	7.4
	≥ 12	18	2	2.1	1.2	90.2	30.1	667,287	4.5
	All ages	20	2	2.1	1.4	116.1	38.7	820,940	4.7
Paratyphoid fever	0 to < 5	0	2	4.1	2.1	–	–	65,904	0.0
	5 to < 12	1	2	3.3	1.5	9.8	3.3	87,749	3.7
	≥ 12	1	2	2.1	1.2	5.0	1.7	667,287	0.3
	All ages	2	2	2.1	1.4	11.6	3.9	8209,40	0.5
Enteric fever	0 to < 5	0	2	4.1	2.1	–	–	65,904	0.0
	5 to < 12	2	2	3.3	1.5	62.4	20.8	87,749	7.4
	≥ 12	20	2	2.1	1.2	400.7	133.6	667,287	5.0
	All ages	22	2	2.1	1.4	333.2	111.1	820,940	5.2

### Validation of the Mahosot Hospital multplier.

Of 336 households responding, 87 (25.9%) reported having a member with fever in the past 3 months. Of those reporting fever, 12 (13.8%) sought care from Mahosot Hospital. The difference between those ‘usually using’ versus ‘actually using’ Mahosot Hospital for fever ≥ 3 days and for any fever, respectively, was statistically significant (48.2% versus 13.8%; *P* < 0.001).

### Incidence calculations.

Of 7,997 blood cultures collected during the surveillance period from patients living in Vientiane, 16 (0.2%) episodes of *Salmonella* Typhi bloodstream infection and six (0.1%) episodes of *Salmonella* Paratyphi A bloodstream infection were identified. *Salmonella* Paratyphi B and C bloodstream infection was not detected (Table 1). After applying hospital, enrollment, and sensitivity multipliers, we estimated the 2015–2017 annual incidence of enteric fever among persons of all ages in Vientiane city to be 5.2 per 100,000, with typhoid fever incidence 4.7 per 100,000 per year and paratyphoid fever incidence 0.5 per 100,000 per year. The incidence was highest in young children aged 5 to < 12 years (Table 1). No enteric fever was confirmed in the age-group 0 through < 5 years, and no paratyphoid fever in the age-group 5 through < 12 years. Further details of incidence calculations are shown in Table 1.

### Sensitivity and uncertainty analysis.

The results of the one-way sensitivity analysis are presented in Table 2. We estimated that the uncertainty interval for annual typhoid fever incidence among persons of all ages ranged from 4.2 to 5.3 cases per 100,000 population, and for paratyphoid fever, it ranged from 0.4 to 0.5 cases per 100,000 population (Table 2).

**Table 2:** Sensitivity analyses and uncertainty of enteric fever incidence, Vientiane, Lao PDR, 2015–2017

		Age (years)	Blood cultures collected	Households	Mahosot % (lower, upper 95% CI)	Mahosot Hospital multiplier (lower, upper 95% CI)	Incidence per 100,000 population
Where would household members usually seek health care if they had fever ≥ 3 days?	Typhoid fever	0 to < 5	558	66	24.2 (14.5–36.4)	4.1 (2.7–7.3)	0.0
		5 to < 12	581	117	30.7 (22.5–39.9)	3.3 (2.5–4.5)	5.8–10.3
		≥ 12	7,506	336	47.9 (42.4–53.4)	2.1 (1.8–2.4)	4.1–5.1
		All ages	8,645	336	48.2 (42.7–53.7)	2.1 (1.4–1.7)	4.2–5.3
	Paratyphoid fever	0 to < 5	558	66	24.2 (14.5–36.4)	4.1 (2.7–7.3)	0
		5 to < 12	581	117	30.7 (22.5–39.9)	3.3 (2.5–4.5)	2.9–5.1
		≥ 12	7,506	336	47.9 (42.4–53.4)	2.1 (1.8–2.4)	0.2–0.3
		All ages	8,645	336	48.2 (42.7–53.7)	2.1 (1.4–1.7)	0.4–0.5
	Enteric fever	0 to < 5	558	66	24.2 (14.5–36.4)	4.1 (2.7–7.3)	0
		5 to < 12	581	117	30.7 (22.5–39.9)	3.3 (2.5–4.5)	5.8–10.3
		≥ 12	7,506	336	47.9 (42.4–53.4)	2.1 (1.8–2.4)	4.5–5.7
		All ages	8,645	336	48.2 (42.7–53.7)	2.1 (1.4–1.7)	4.6–5.8

## DISCUSSION

We found that the incidence of typhoid fever, paratyphoid fever, and enteric fever overall in Vientiane in 2015–2017 was low. A recent systematic review of global typhoid fever incidence studies showed that annual incidence in studies carried out at sites in Asia in the period 2000–2013 ranged from 15 per 100,000 population in China^22^ to 1,279 per 100,000 population in India.^23^ The overall annual typhoid fever incidence across Asian studies in this systematic review was 190 per 100,000 population.^24^ Furthermore, a sister study to ours performed in Yangon, Myanmar, for the period 2015–2016 estimated annual typhoid fever and paratyphoid fever incidence there at 391 and 107 per 100,000 population, respectively, albeit with wide uncertainty bounds.^13^ Of note, in Vientiane *Salmonella* Typhi was isolated from blood cultures considerably more frequently in the period 2000–2004^5^ than in 2015–2017, suggesting that typhoid fever incidence is likely to be considerably lower in the contemporary time period compared with 15 years earlier. However, the lack of a healthcare utilization survey from the period 2000–2004 precluded an incidence estimation for the earlier period.

It is increasingly recognized that typhoid fever incidence varies considerably in time at the same location and also by place, including within the same country.^24^ Therefore, the low typhoid and paratyphoid fever incidence observed in Vientiane 2015–2017 does not mean that incidence will remain low in the future nor that enteric fever was uncommon elsewhere in the country during the same period. The variation in endemic typhoid fever incidence makes national policy decisions based on data from a single site in a limited time period challenging. Although data for the Lao PDR were lacking for 1990, by 2015, it was estimated that 94% and 86% of the urban population had access to improved sanitation and water, respectively.^25^ This high level of coverage may be the result of recent economic development. However, among the much larger Lao PDR rural population in 2015, only 56% and 69% were reported to have access to improved sanitation and water, respectively.^25^ Therefore, despite the present low incidence of enteric fever in Vientiane, it is reasonable to surmise that risk for transmission of typhoidal *Salmonella* persists, especially in areas underserved by improved sanitation and water infrastructure.

Neither *Salmonella* Typhi nor *Salmonella* Paratyphi A was isolated from blood cultures of infants and young children at Mahosot Hospital in the period 2015–2017 nor was *Salmonella* Paratyphi A isolated from older children. Obtaining adequate blood culture volumes may be more difficult from infants and young children than from older children and adults. Because of the low magnitude of *Salmonella* Typhi bacteremia, detection is less likely if smaller volumes of blood are collected. This limitation is partially offset by the higher magnitude of *Salmonella* Typhi bacteremia observed in younger patients.^26^ However, it is well established that typhoid fever incidence varies by age with respect to the overall level of incidence. For example, typhoid incidence is usually highest in infants and young children in high-incidence settings but more evenly distributed across age-groups where incidence is low. Therefore, it is possible that the lack of enteric fever identified in younger age-groups reflects in part the epidemiology of the disease when transmission intensity is lower.

Our study had a number of limitations. Although hybrid surveillance is a well-established method for estimating disease incidence, the incidence estimates are sometimes based on a small number of crude cases. This was the case in our study, but in light of data from earlier periods likely reflects low enteric fever incidence in Vientiane during the study period. There are a range of healthcare facilities in Vientiane. Our healthcare utilization survey showed that Mahosot Hospital was not a particularly common place to seek care for prolonged fever, despite the high reported preference. However, the hospital multiplier was not as large as for our sister study in Yangon, Myanmar,^13^ and thus, uncertainty around incidence estimates was not as wide. Although almost half of community members reported that they would use Mahosot Hospital in the event of fever for ≥ 3 days, only 13.8% of those with fever during the previous 3 months actually went to Mahosot Hospital. This difference likely reflects some form of reporting bias during the healthcare utilization survey that might be associated with underestimation of typhoid and paratyphoid fever incidence.

In conclusion, we found that typhoid and paratyphoid fever incidence in Vientiane, Lao PDR, was low in the period 2015–2017. Earlier studies of bloodstream infections at Mahosot Hospital suggest that incidence was likely considerably higher in previous years. However, data on access to improved sanitation and water facilities in Lao PDR suggest that risk factors for enteric fever transmission persist. In light of variation in typhoid fever that occurs in place and time, additional data from other parts of Lao PDR would be helpful to support decisions about typhoid conjugate vaccine introduction and ongoing surveillance in Vientiane is warranted.
